# Supplementary material for: A flexible age-dependent, spatially-stratified predictive model for the spread of COVID-19, accounting for multiple viral variants and vaccines
Source: PLoS One. 2023 Jan 20;18(1):e0277505. doi: 10.1371/journal.pone.0277505 (PMC9858464; doi:10.1371/journal.pone.0277505)
Supplement: S1 Table — (PDF) [file pone.0277505.s003.pdf]

S1 Table. Age-stratified population size of Germany (GER).

Parameter	Description	GER
N	Total population size	83 787 388
N_1	No. of inds. in age group 1 (0-5 years)	3 969 138
N_2	No. of inds. in age group 2 (6-19 years)	11 365 436
N_3	No. of inds. in age group 3 (20-59 years)	43 730 684
N_4	No. of inds. in age group 4 (60+ years)	24 722 130

Parameters describing the population sizes. Abbreviations: inds. ... individuals.
