# Supplementary material for: A flexible age-dependent, spatially-stratified predictive model for the spread of COVID-19, accounting for multiple viral variants and vaccines
Source: PLoS One. 2023 Jan 20;18(1):e0277505. doi: 10.1371/journal.pone.0277505 (PMC9858464; doi:10.1371/journal.pone.0277505)
Supplement: S2 Table — (PDF) [file pone.0277505.s004.pdf]

S2 Table. Timeline of contact reduction measures chosen for the simulations of Germany.

Parameter	Description	Value
t_{Dist_1}	First "hard lockdown" (general distancing) starts	40
t_{Dist_2}	First "hard lockdown" ends and first "relief period" starts	85
t_{Dist_3}	"Relief period" continues as summer vacation starts	97
t_{Dist_4}	"Relief period" ends as summer vacation ends	170
t_{Dist_5}	Start of "weak measures" after summer vacation	190
t_{Dist_6}	"Weak measures" ends and "soft lockdown" starts	245
t_{Dist_7}	"Soft lockdown" ends and Christmas "hard lockdown" starts	280
t_{Dist_8}	"Hard lockdown" gets stricter before Christmas	303
t_{Dist_9}	"Hard lockdown" ends and "soft lockdown" starts	355
$t_{\text{Dist}_{10}}$	Start of "emergency brake" on April 25	425
$t_{\text{Dist}_{11}}$	"emergency brake" ends	490
$t_{\text{Dist}_{12}}$	"Soft lockdown" and 3G rule starts	540
$t_{\text{Dist}_{13}}$	"Soft lockdown" ends and"relief period" and 2G rule starts	621
$t_{\text{Dist}_{14}}$	"Hypothetical school closures" starts	636
$t_{\text{Dist}_{15}}$	"Hypothetical school closures" ends	850
