# Supplementary material for: A flexible age-dependent, spatially-stratified predictive model for the spread of COVID-19, accounting for multiple viral variants and vaccines
Source: PLoS One. 2023 Jan 20;18(1):e0277505. doi: 10.1371/journal.pone.0277505 (PMC9858464; doi:10.1371/journal.pone.0277505)
Supplement: S4 Table — (PDF) [file pone.0277505.s006.pdf]

S4 Table. Parameters describing incidence-based contact reductions for emergency brake conditions in Germany.

Incidence thresholds*	Home	Home(Old)	Others	Others(Old)	School	Work
<10	0%	0%	50%	10%	25%	30%
10	25%	5%	60%	20%	50%	55%
50	30%	10%	85%	60%	75%	65%
100	40%	20%	95%	75%	75%	85%
180	40%	35%	95%	75%	100%	85%

*Incidence values (per 100 000 individuals) triggering contact reductions.
