# Supplementary material for: A flexible age-dependent, spatially-stratified predictive model for the spread of COVID-19, accounting for multiple viral variants and vaccines
Source: PLoS One. 2023 Jan 20;18(1):e0277505. doi: 10.1371/journal.pone.0277505 (PMC9858464; doi:10.1371/journal.pone.0277505)
Supplement: S5 Table — (PDF) [file pone.0277505.s007.pdf]

S5 Table. Parameters describing the vaccination rate.

Parameter	Description		Value
	Rate at which inds. get vaccinated with:		
$\nu_{1,l}^{(1)}$	vaccine 1 in age gr. 1		0
$\nu_{2,l}^{(1)}$	vaccine 1 in age gr. 2	$t \in [0, 590)$	0
		$t \in [590, 850]$	1/170
$\nu_{3,l}^{(1)}$	vaccine 1 in age gr. 3	$t \in [0, 430)$	0
		$t \in [430, 850]$	1/240
$\nu_{4,l}^{(1)}$	vaccine 1 in age gr. 4	$t \in [0, 360)$	0
		$t \in [360, 400)$	1/280
		$t \in [400, 490)$	1/160
		$t \in [490, 850]$	1/140
$\nu_{1,l}^{(2)}$	vaccine 2 in age gr. 1		0
$\nu_{2,l}^{(2)}$	vaccine 2 in age gr. 2		0
$\nu_{3,l}^{(2)}$	vaccine 2 in age gr. 3	$t \in [0, 430)$	0
		$t \in [430, 850]$	1/240
$\nu_{4,l}^{(2)}$	vaccine 2 in age gr. 4	$t \in [0, 400)$	0
		$t \in [400, 490)$	1/400
		$t \in [490, 850]$	1/340
$\nu_{1,l}^{(3)}$	vaccine 3 in age gr. 1		0
$\nu_{2,l}^{(3)}$	vaccine 3 in age gr. 2		0
$\nu_{3,l}^{(3)}$	vaccine 3 in age gr. 3	$t \in [0, 490)$	0
		$t \in [490, 850]$	1/240
$\nu_{4,l}^{(3)}$	vaccine 3 in age gr. 4	$t \in [0, 490)$	0
		$t \in [490, 850]$	1/400

Parameters describing vaccination rates and their values used in the simulations. Abbreviations: gr. . . . group.
