# Supplementary material for: A flexible age-dependent, spatially-stratified predictive model for the spread of COVID-19, accounting for multiple viral variants and vaccines
Source: PLoS One. 2023 Jan 20;18(1):e0277505. doi: 10.1371/journal.pone.0277505 (PMC9858464; doi:10.1371/journal.pone.0277505)
Supplement: S6 Table — (PDF) [file pone.0277505.s008.pdf]

S6 Table. Parameters describing disease progression.

Parameter	Description	Value
n_E	No. of latent sub-states (Erlang states)	5
n_P	No. of prodromal sub-states (Erlang states)	5
n_I	No. of fully-infectious sub-states (Erlang states)	5
n_L	No. of late-infectious sub-states (Erlang states)	5
	Average duration of:	
D_E	latent period	3.5 days
D_P	prodromal period	1 day
D_I	fully-infectious period	5 days
D_L	late-infectious period	5 days
	Transition rate of ($a = 1, \ldots, 4$, $m = 1, 2, 3$):	
$\varepsilon_{a,m}$	latent sub-states	$n_{\mathrm{E}}/D_{\mathrm{E}}$
$\varphi_{a,m}$	prodromal sub-states	$n_{\mathrm{P}}/D_{\mathrm{P}}$
$\gamma_{a,m}$	fully-infectious sub-states	$n_{\mathrm{I}}/D_{\mathrm{I}}$
$\delta_{a,m}$	late-infectious sub-states	$n_{\mathrm{L}}/D_{\mathrm{L}}$
	For $a = 1, \ldots, 4$:	
$\alpha_a^{(1)}, \alpha_a^{(2)}, \alpha_a^{(3)}$	average waiting time for the outcome of the vaccines	$1/30, 1/50, 1/15$
