# Supplementary material for: A flexible age-dependent, spatially-stratified predictive model for the spread of COVID-19, accounting for multiple viral variants and vaccines
Source: PLoS One. 2023 Jan 20;18(1):e0277505. doi: 10.1371/journal.pone.0277505 (PMC9858464; doi:10.1371/journal.pone.0277505)
Supplement: S7 Table — (PDF) [file pone.0277505.s009.pdf]

S7 Table. Parameters describing vaccination outcome and immunity.

Parameters	Description ($m = 1, 2, 3$)			Value
	Fraction of susceptibles that:	v: 1	2	3
$f_S^{(\mathrm{NI},v)}$	fail to immunize upon vaccination	0.05	0.05	0.05
$f_S^{(\mathrm{PI},v)}$	develop partial immunity upon vaccination	0.70	0.65	0.65
$f_S^{(\mathrm{Im},v)}$	develop full immunity after vaccination	0.25	0.30	0.30
	Fraction of latent individuals that:			
$f_E^{(\mathrm{NI},v)}$	fail to immunize after vaccination	0.50	0.50	0.50
$f_E^{(\mathrm{PI},v)}$	develop partial immunity after vaccination	0.50	0.50	0.50
$f_E^{(\mathrm{Im},v)}$	develop full immunity after vaccination	0.00	0.00	0.00
	Fraction of prodromal individuals that:			
$f_P^{(\mathrm{NI},v)}$	fail to immunize after vaccination	0.50	0.50	0.50
$f_P^{(\mathrm{PI},v)}$	develop partial immunity after vaccination	0.50	0.50	0.50
$f_P^{(\mathrm{Im},v)}$	develop full immunity after vaccination	0.00	0.00	0.00
	Fraction of fully-infectious individuals that:			
$f_I^{(\mathrm{NI},v)}$	fail to immunize after vaccination	0.50	0.50	0.50
$f_I^{(\mathrm{PI},v)}$	develop partial immunity after vaccination	0.50	0.50	0.50
$f_I^{(\mathrm{Im},v)}$	develop full immunity after vaccination	0.00	0.00	0.00
	Fraction of late-infectious individuals that:			
$f_L^{(\mathrm{NI},v)}$	fail to immunize after vaccination	0.50	0.50	0.50
$f_L^{(\mathrm{PI},v)}$	develop partial immunity after vaccination	0.50	0.50	0.50
$f_L^{(\mathrm{Im},v)}$	develop full immunity after vaccination	0.00	0.00	0.00
	Fraction by which partial immunity reduces transmissibility in the:			
$p_P^{(m,v)}$	prodromal period	0.66	0.70	0.66
$p_I^{(m,v)}$	fully-infectious period	0.66	0.70	0.66
$p_L^{(m,v)}$	late-infectious period	0.66	0.70	0.66
	Susceptibility reduced by partial immunity against:			
$g(1, v)$	variant 1	0.50	0.50	0.55
$g(2, v)$	variant 2	0.60	0.60	0.60
$g(3, v)$	variant 3	0.60	0.60	0.60
$h(m, v)$	Susceptibility reduced by full immunity			0
