# Supplementary material for: A flexible age-dependent, spatially-stratified predictive model for the spread of COVID-19, accounting for multiple viral variants and vaccines
Source: PLoS One. 2023 Jan 20;18(1):e0277505. doi: 10.1371/journal.pone.0277505 (PMC9858464; doi:10.1371/journal.pone.0277505)
Supplement: S8 Table — (PDF) [file pone.0277505.s010.pdf]

S8 Table. Parameters describing disease severity and mortality.

Parameters	Description ($m = 1, 2, 3$)				Value
	Fraction of sympt. inds. in:	m:	1	2	3
$f_{\mathrm{Sick}}^{(1,m)}$	age gr. 1		0.15	0.15	0.15
$f_{\mathrm{Sick}}^{(2,m)}$	age gr. 2		0.30	0.30	0.30
$f_{\mathrm{Sick}}^{(3,m)}$	age gr. 3		0.65	0.65	0.65
$f_{\mathrm{Sick}}^{(4,m)}$	age gr. 4		0.70	0.70	0.70
	Fraction of sympt. inds. who die in:	m:	1	2	3
$f_{\mathrm{dead}}^{(1,m)}$	age gr. 1		0.0001	0.0002	0.0004
$f_{\mathrm{dead}}^{(2,m)}$	age gr. 2		0.0001	0.0002	0.0004
$f_{\mathrm{dead}}^{(3,m)}$	age gr. 3		0.0010	0.0011	0.0012
$f_{\mathrm{dead}}^{(4,m)}$	age gr. 4		0.2400	0.2400	0.2400
	Fraction of sympt. inds. with part. immunity in:	v:	1	2	3
$f_{\mathrm{Sick}}^{(\mathrm{PI},1,1,v)}$	age gr. 1 infected with variant 1		0.10	0.11	0.10
$f_{\mathrm{Sick}}^{(\mathrm{PI},1,2,v)}$	age gr. 1, infected with variant 2		0.11	0.12	0.11
$f_{\mathrm{Sick}}^{(\mathrm{PI},1,3,v)}$	age gr. 1, infected with variant 3		0.12	0.13	0.12
$f_{\mathrm{Sick}}^{(\mathrm{PI},2,1,v)}$	age gr. 2, infected with variant 1		0.25	0.22	0.25
$f_{\mathrm{Sick}}^{(\mathrm{PI},2,2,v)}$	age gr. 2, infected with variant 2		0.26	0.23	0.26
$f_{\mathrm{Sick}}^{(\mathrm{PI},2,3,v)}$	age gr. 2, infected with variant 3		0.27	0.24	0.27
$f_{\mathrm{Sick}}^{(\mathrm{PI},3,1,v)}$	age gr. 3, infected with variant 1		0.55	0.60	0.55
$f_{\mathrm{Sick}}^{(\mathrm{PI},3,2,v)}$	age gr. 3, infected with variant 2		0.56	0.61	0.56
$f_{\mathrm{Sick}}^{(\mathrm{PI},3,3,v)}$	age gr. 3, infected with variant 3		0.57	0.62	0.57
$f_{\mathrm{Sick}}^{(\mathrm{PI},4,1,v)}$	age gr. 4, infected with variant 1		0.60	0.60	0.60
$f_{\mathrm{Sick}}^{(\mathrm{PI},4,2,v)}$	age gr. 4, infected with variant 2		0.60	0.60	0.60
$f_{\mathrm{Sick}}^{(\mathrm{PI},4,3,v)}$	age gr. 4, infected with variant 3		0.60	0.60	0.60
	Fraction of inds. with part. immunity who die in:	v:	1	2	3
$f_{\mathrm{Dead}}^{(\mathrm{PI},1,1,v)}$	age gr. 1, infected with variant 1		0.00	0.00	0.00
$f_{\mathrm{Dead}}^{(\mathrm{PI},1,2,v)}$	age gr. 1, infected with variant 2		0.00	0.00	0.00
$f_{\mathrm{Dead}}^{(\mathrm{PI},1,3,v)}$	age gr. 1, infected with variant 3		0.00	0.00	0.00
$f_{\mathrm{Dead}}^{(\mathrm{PI},2,1,v)}$	age gr. 2, infected with variant 1		0.5e-5	5.5e-6	0.5e-5
$f_{\mathrm{Dead}}^{(\mathrm{PI},2,2,v)}$	age gr. 2, infected with variant 2		1.0e-5	1.6e-5	1.0e-5
$f_{\mathrm{Dead}}^{(\mathrm{PI},2,3,v)}$	age gr. 2, infected with variant 3		2.0e-5	2.5e-5	2.0e-5
$f_{\mathrm{Dead}}^{(\mathrm{PI},3,1,v)}$	age gr. 3, infected with variant 1		5.5e-5	6.0e-5	5.5e-5
$f_{\mathrm{Dead}}^{(\mathrm{PI},3,2,v)}$	age gr. 3, infected with variant 2		5.5e-5	6.0e-5	5.5e-5
$f_{\mathrm{Dead}}^{(\mathrm{PI},3,3,v)}$	age gr. 3, infected with variant 3		5.5e-5	6.0e-5	5.5e-5
$f_{\mathrm{Dead}}^{(\mathrm{PI},4,1,v)}$	age gr. 4, infected with variant 1		0.0040	0.0045	0.0040
$f_{\mathrm{Dead}}^{(\mathrm{PI},4,2,v)}$	age gr. 4, infected with variant 2		0.0040	0.0045	0.0040
$f_{\mathrm{Dead}}^{(\mathrm{PI},4,3,v)}$	age gr. 4, infected with variant 3		0.0040	0.0045	0.0040

Summary of parameters describing disease severity and mortality. Abbreviations: inds. ... individuals; sympt. ... symptomatic.
