# Supplementary material for: A flexible age-dependent, spatially-stratified predictive model for the spread of COVID-19, accounting for multiple viral variants and vaccines
Source: PLoS One. 2023 Jan 20;18(1):e0277505. doi: 10.1371/journal.pone.0277505 (PMC9858464; doi:10.1371/journal.pone.0277505)
Supplement: S9 Table — (PDF) [file pone.0277505.s011.pdf]

S9 Table. Variables describing initial values of individuals in non-infected compartments.

Name	Description	Initial Values
	Vaccinable suscept. in:	
$S_1^{(\mathrm{U})}(0)$	age group 1	0
$S_2^{(\mathrm{U})}(0)$	age group 2	4 091 557
$S_3^{(\mathrm{U})}(0)$	age group 3	37 171 081
$S_4^{(\mathrm{U})}(0)$	age group 4	23 486 024
	For $a = 1, \ldots, 4$, $v = 1, 2, 3$, $m = 1, 2, 3$:	
$S_a^{(\mathrm{V},v)}(0)$	vacc. suscept. with pending vaccine outcome	0
$S_a^{(\mathrm{NI})}(0)$	unvaccinable & unsuccessfully immune inds.	0
$S_a^{(\mathrm{PI},v)}(0)$	vacc. suscept. who developed partial immunity	0
$R_a^{(\mathrm{Im},v)}(0)$	vacc. suscept. with full immunity (against at least one variant)	0
$R_a^{(\mathrm{Inf},m)}(0)$	Recovered or fully immune individuals	0
$D_a^{(m)}(0)$	Individuals who die from COVID-19	0

Summary of non-infected compartments and their initial values. Abbreviations: inds. . . . individuals; suscept. . . . susceptibles; vacc. . . . vaccinated.
