# Supplementary material for: A flexible age-dependent, spatially-stratified predictive model for the spread of COVID-19, accounting for multiple viral variants and vaccines
Source: PLoS One. 2023 Jan 20;18(1):e0277505. doi: 10.1371/journal.pone.0277505 (PMC9858464; doi:10.1371/journal.pone.0277505)
Supplement: S10 Table — (PDF) [file pone.0277505.s012.pdf]

S10 Table. Variables describing initial values of individuals in infected compartments.

Name	Description ($a = 1, \ldots, 4$, $m = 1, 2, 3$, $v = 1, 2, 3$)	Initial Values
	For $k = 1, \ldots, n_E$:	
$E_{k,a}^{(\mathrm{U},m)}(0)$	vaccinable lat. infected inds.	0
$E_{k,a}^{(\mathrm{V},m,v)}(0)$	vaccinated lat. infected inds. with pending vaccine outcome	0
$E_{k,a}^{(\mathrm{NI},m)}(0)$	unvaccinable & unsuccessfully lat. infected inds.	0
$E_{k,a}^{(\mathrm{PI},m,v)}(0)$	vaccinated lat. infected inds. who developed partial immunity	0
	For $k = 1, \ldots, n_P$:	
$P_{k,a}^{(\mathrm{U},m)}(0)$	vaccinable prodromal inds.	0
$P_{k,a}^{(\mathrm{V},m,v)}(0)$	vaccinated prodromal inds. with pending vaccine outcome	0
$P_{k,a}^{(\mathrm{NI},m)}(0)$	unvaccinable & unsuccessfully immune prodromal inds.	0
$P_{k,a}^{(\mathrm{PI},m,v)}(0)$	vaccinated prodromal inds. who developed partial immunity	0
	No. of initial infs. of vaccinable inds. in:	
$I_{1,1}^{(\mathrm{U},m)}(0)$	age gr. 1	0
$I_{1,2}^{(\mathrm{U},m)}(0)$	age gr. 2	10
$I_{1,3}^{(\mathrm{U},m)}(0)$	age gr. 3	50
$I_{1,4}^{(\mathrm{U},m)}(0)$	age gr. 4	5
	For $k = 2, \ldots, n_I$: vaccinable fully-infectious inds. in:	
$I_{k,1}^{(\mathrm{U},m)}(0)$	age gr. 1	0
$I_{k,2}^{(\mathrm{U},m)}(0)$	age gr. 2	0
$I_{k,3}^{(\mathrm{U},m)}(0)$	age gr. 3	0
$I_{k,4}^{(\mathrm{U},m)}(0)$	age gr. 4	0
$I_{k,a}^{(\mathrm{V},m,v)}(0)$	fully-infectious inds. with pending vaccine outcome	0
$I_{k,a}^{(\mathrm{NI},m)}(0)$	unvaccinable & unsuccessfully immune fully-infectious inds.	0
$I_{k,a}^{(\mathrm{PI},m,v)}(0)$	vaccinated fully-infectious inds. who developed partial immunity	0
	For $k = 2, \ldots, n_L$:	
$L_{k,a}^{(\mathrm{U},m)}(0)$	vaccinable late-infectious inds.	0
$L_{k,a}^{(\mathrm{V},m,v)}(0)$	late-infectious inds. with pending vaccine outcome	0
$L_{k,a}^{(\mathrm{NI},m)}(0)$	unvaccinable & unsuccessfully immune late-infectious inds.	0
$L_{k,a}^{(\mathrm{PI},m,v)}(0)$	vaccinated late-infectious inds. who developed partial immunity	0

Summary of infected compartments and their initial values chosen for simulation. Abbreviations: inds. . . . individuals; lat. . . . latently; gr. . . . group.
