# Supplementary material for: A flexible age-dependent, spatially-stratified predictive model for the spread of COVID-19, accounting for multiple viral variants and vaccines
Source: PLoS One. 2023 Jan 20;18(1):e0277505. doi: 10.1371/journal.pone.0277505 (PMC9858464; doi:10.1371/journal.pone.0277505)
Supplement: S11 Table — (PDF) [file pone.0277505.s013.pdf]

S11 Table. Parameters describing external-, seasonal-factors, control measures, and contagiousness.

Parameter	Definition	Value/Eq.
λ_{Ext}	Infections from outside of the population	30/day
$\bar{R}_0^{(1)}, \bar{R}_0^{(2)}, \bar{R}_0^{(3)}$	Annual average basic reproduction number	3.0, 3.9, 5.46
A	Amplitude of the seasonal fluctuation in R_0	0.43
$t_{R_{0_{\max}}}$	Day when R_0 reaches its maximum	300
$Q_{\max}$	Maximum capacity of isolation units per 10,000	200
t_{Iso_2}	Day case isolation measures start	10
t_{Iso_1}	Day case isolation measures end	850
$t_{\text{Mut}}^{(1)}, t_{\text{Mut}}^{(2)}, t_{\text{Mut}}^{(3)}$	Day mutations are introduced into the population	-20, 290, 475
f_{Iso}	Fraction of inf. inds. who are isolated	58%
p_{Home}	Contact reduction in home isolation	75%
c_P	Relative contagiousness in prodromal period	0.5
c_I	Relative contagiousness in fully-infectious phase	1
c_L	Relative contagiousness in late-infectious phase	0.5
$\beta_P(t)$	Seasonally varying effective contact rate of prodromal inds.	cf. eq. 8a
$\beta_I(t)$	Seasonally varying effective contact rate of fully-infectious inds.	cf. eq. 8b
$\beta_L(t)$	Seasonally varying effective contact rate of late-infectious inds.	cf. eq. 8c

Parameter	Day when vaccination campaigns start for:	v: 1	2	3
$t_{\text{Vacc}}^{(1,v)}$	age group 1	850	850	850
$t_{\text{Vacc}}^{(2,v)}$	age group 2	490	850	850
$t_{\text{Vacc}}^{(3,v)}$	age group 3	400	400	430
$t_{\text{Vacc}}^{(4,v)}$	age group 4	310	360	430
