# Supplementary material for: A flexible age-dependent, spatially-stratified predictive model for the spread of COVID-19, accounting for multiple viral variants and vaccines
Source: PLoS One. 2023 Jan 20;18(1):e0277505. doi: 10.1371/journal.pone.0277505 (PMC9858464; doi:10.1371/journal.pone.0277505)
Supplement: S2 Fig — (PDF) [file pone.0277505.s015.pdf]

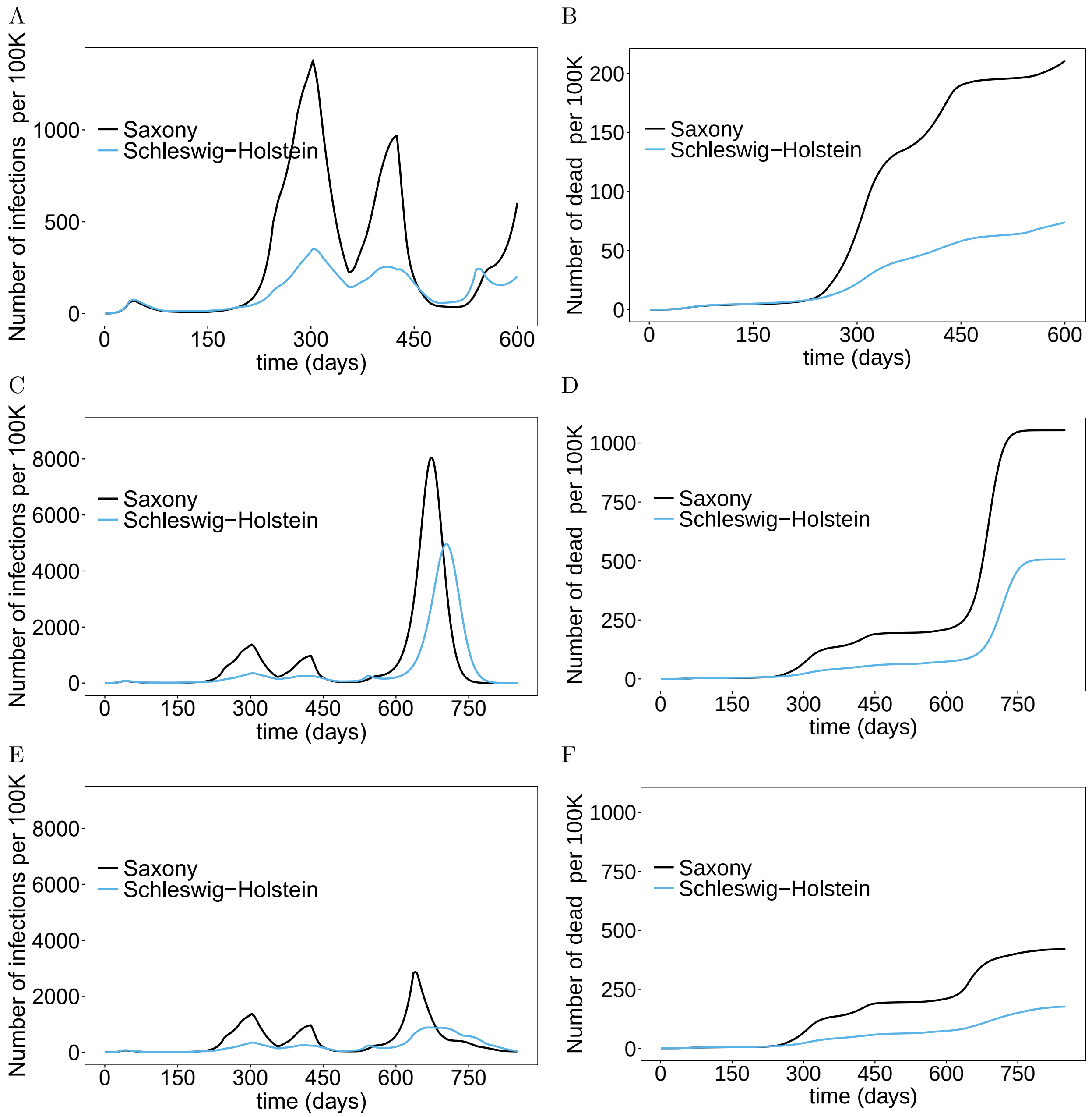

S2 Fig. Effect of emergency brake – two locations model: (A) Shown are the total numbers of infected individuals assuming the same contact reductions after November 7, 2021 as one year earlier, with and without the weather adjustments in 2021. (B) Shown are the corresponding deaths. The parameters used for the simulations are listed in S1 Table - S11 Table and in the tables in S1 Simulation results for Saxony and Schleswig-Holstein.
