# Supplementary material for: A flexible age-dependent, spatially-stratified predictive model for the spread of COVID-19, accounting for multiple viral variants and vaccines
Source: PLoS One. 2023 Jan 20;18(1):e0277505. doi: 10.1371/journal.pone.0277505 (PMC9858464; doi:10.1371/journal.pone.0277505)
Supplement: Attachment — Submitted filename: Reply to Reviewers.pdf [file pone.0277505.s016.pdf]

Reply to Reviewers

Reviewer #1: This is a comprehensive and flexible COVID-19 transmission model with a user front-end and open access code. While the authors state in the last line of the Discussion that temporary immunity can be implemented, the authors are encouraged to state if and when this improvement will be made to capture naturally acquired and vaccine-induced waning immunity. As well, hospital admissions should be reported as part of these findings.

MAJOR COMMENTS
1. Abstract, Methods and findings: the authors are encouraged to report the projections for the reduction in hospital admissions (i.e., health system burden and health system stress) in addition to the effect on infections and mortality.

> This is doubtlessly an important point, but unfortunately an almost impossible task to achieve due to practical issues. This has several reasons. First, healthcare systems and policies are rather heterogeneous across countries and even within countries. For instance, in some countries like Peru, only limited amounts of ventilators are available, in France patients aged 69+ would not receive ECMO-therapies as wouldn't patients that have been on the ventilator in ICU for more than 14 days or so (this is the reason why France has fantastic survival rates of ECMO patients), while in Germany pretty much everybody would receive ECMO-therapy if necessary. In fact, in Germany, not all available ICU beds could be filled, because the ICU teams were overwhelmed with treating the labor-intensive ECMO patients. This changed drastically with Omicron. ECMO-therapies are rarely used now, but incidence is so high, that the increase in sick leaves of medical personnel is threatening ICU capacities. Another practical issue, which arises specifically in Germany, is the lack of data collected in hospitals. Often the vaccination status is unclear (not to speak of the vaccine products they were vaccinated with). Moreover, it is not even registered whether patients are admitted to hospital because of COVID or whether they are infected in the hospital with COVID. While it makes little difference for the hospital, because the effort to isolate patients in COVID-ward is always the same, it is an important distinction to assess the effectiveness of vaccines. Another aspect is changing policies. In the first wave, there was a tendency to treat patients too soon on the ventilator, which was avoided in subsequent waves. Moreover, with therapies becoming available, e.g., antibody-based therapies, Paxlovid, etc., the effect of the pandemic on the healthcare system is rapidly changing. The long story short, to capture the actual implications in terms of hospital admission (with or because of COVID) is country specific and would require a lot of detailed in depth knowledge. As a proxy, the hospitalizations correlate with the number of deaths.
> If fine-grained information is available, it is possible to derive the implications from the model solution (i.e., for the infected individuals in the respective model compartments). However, we feel the hospitalizations correlate with the deaths and everything more involved would require in-depth knowledge of healthcare experts and a refined fine structured model to derive the implications from the model solution. Such an endeavor is beyond the scope of our ms and beyond our expertise.

MINOR COMMENTS
2. Abstract, Methods and findings: the section "and predicted the future dynamics of the epidemic under different interventions" could be revised to "and future dynamics of the epidemic under different interventions were predicted."

> Appreciated and Done!

3. Abstract, Methods and findings: the authors should consider indicating when they predict a strong epidemic wave in Germany, i.e., at least in which season of 2022 this wave is predicted.

> Unfortunately, the ms went to revision very late. It was submitted before the Omicron variant occurred. This variant was a game-changer in several respects. Namely, reinfection can no longer be neglected - hence the model assumption of permanent immunity is no longer justified. This requires substantial changes in the original model. We are working and using already a

prototype of such a model, however, the changes we had to make are not just simple adjustments, rather it is a whole new model. Factors like immune waning are dominating the dynamics under the different Omicron variants. In general, it can be said that waves will occur seasonally, but it is pointless to predict the future waves with a model that is on the knowledge-level of the time of initial submission in November 2021. Actually, the predictions of the model for the Delta-wave in Germany until January 2022 were pretty accurate. However, Omicron changed the picture.

4. Abstract, Methods and findings: could the authors be more specific about how they define ‘mandatory vaccination’ (i.e., dose number, target group, coverage, and timing.)

This is important! More information explaining what is meant by ``mandatory vaccination´´ was added.

5. Abstract, Conclusions: the aspect of deriving economic collateral damages is only introduced in the conclusion as a feature of the model, and readers will need to have further elaboration as to what this encompasses. For example, does this tool consider labor force loss, border closure implications on trade, effect on domestic growth, impact on tax revenues, increased expenditures on health and other areas, the decline in revenue, etc.?

The model does not include this information on a fine-grained scale, obviously. However, the model structure is fine-grained enough to provide the necessary input to derive things like losses in labor force etc. This has to be done by the reader. We believe that the whole model and its description is already complex. Adding more complexity would only cause confusion. We rephrased the sentence a bit, to make this clearer.

6. Introduction, line 1: do the authors think it is fair to state the COVID-19 vaccines were developed to bring a ‘quick end’ to the pandemic? Developers and planners were likely well aware that restrictions around supply and demand, rollout, and vaccine efficacy in light of emerging new variants with changing would unfortunately not result in a quick end of a global pandemic. Perhaps vaccines were developed to reduce the impact and burden of the pandemic and potentially towards the eventual end of the pandemic.

We believe it is a fair statement to say. With predictions based on this model, we were covered in numerous media formats in Germany. The public discussion, especially regarding the vaccines, evolves exactly around this point. Clearly, researchers were more careful in their predictions, but the introduction is not just there to put our work in a scientific context but also a broader context. Moreover, looking at the clinical studies of BioNTech, there were hopes that the vaccine induced longer-lasting immunity. Nevertheless, we rephrased the sentence.

7. Introduction, lines 3 and 4: the sentence ‘With BNT162b2 and mRNA-1273, two mRNA-based vaccines received approval for emergency or full use and were widely deployed’ needs to be reworded, e.g., BNT162b2 and mRNA-1273, are two mRNA-based vaccines the received approval for emergency or full use and were widely deployed’. Also, were these vaccines widely deployed evenly across all world continents? In the next sentence, are these 2 vaccines included in the set of 23 vaccines? The authors may consider listing the types of vaccines (mRNA, vector (disabled adenovirus), protein subunit, yeast fermentation). The section ‘for teenagers and children’, should likely read something like ‘for administration among teenagers and children’.

We rephrased the corresponding paragraph. We avoided going into the details of worldwide vaccination deployment, because it is a highly controversial discussion. China and Russia have their own policies. In Africa, vaccination deployment seems low, but considering the demographics in many of its countries, the picture changes. The deployment of the mRNA vaccines in North America and Europe are sufficient to justify the term “widespread deployment”.

8. Introduction, line 7: the authors may consider reframing their statement that it is ‘unclear to which extent the various vaccines protect from infection and transmission’. While the global clinical community have not been able to track the efficacy of the newly developed COVID-19 vaccines over relatively long periods, as clinical trial evidence has been published, as stated in the next sentence, i.e., empirical

evidence suggests, perhaps this could be framed as evidence needs further follow-up or examination or validation. Unclear does not seem to be the appropriate term to me. Duration of protection (i.e., decay) being unclear seems to be a fair assessment.

We agree with the reviewer and replaced “unclear” with “not thoroughly assessed”. Moreover, a reference “PeirisLeung2020” was added to clarify our point. Note, however, the limited applicability of clinical studies. The original clinical study of BioNTech suggested that the vaccine protects from infection. However, the limitation was, that it was conducted when incidence was low, and participants had only few infectious contacts. If the vaccine reduces the probability of transmission during a potentially infectious event, the number of such events necessary for successful transmission follows a geometric distribution. In low-incidence settings, this can erroneously suggest a higher protection from infection.

9. Introduction, line 20: the statement that vaccination coverage eventually saturated is quite broad and unspecific. Is there a certain coverage level(s) and timeline of this saturation?

We merged the sentence with the next, which gives the explanation why.

10. Introduction, lines 42-44: this sentence could be slightly reworded to something like ‘Due to human contact behaviour being age and location dependent, a sufficient age- and location-dependent vaccination coverage level is important for containing the spread of the virus.’

Done.

11. Introduction, line 56: specify that this is an individual-based model.

We introduce a compartmental model, not an individual-based model. We pointed that out even more now.

12. Introduction, line 61: the authors may wish to elaborate on the statement that vaccine efficacy is location dependent because of location-specific contact dynamics, and not because efficacy alone differs by location.

This is a good idea. We elaborated it more, but kept it to a minimum, as a detailed explanation would be too lengthy at this point.

13. Methods, line 77: does the model also captured risk by age-structure in the form of co-morbidities?

We clarify now that co-morbidities are subsumed by the age-specific proportions of severe cases and age-specific mortality rates.

14. Methods, line 81: add a comma before ‘etc.’ It may be useful to include if the model captures treatment interventions within ‘etc.’, for example antivirals like paxlovid.

Comm was added. Treatment interventions are not explicitly modeled. At the time the model was developed, no effective COVID chemotherapy was available, and treatment mainly based on steroid use, remdesivir, antibody therapies. The effect of Paxlovid could be subsumed by age-dependent mortality rates and fractions of severe infections. This is not explicitly modeled. It is easy to adapt the programming code. Another possibility is to break the simulation in two and change the above-mentioned parameters. The practical problem arising is three-fold: first, there is no sufficient fine-grained data to ascertain the age-dependent effect of Paxlovid in combination with vaccines, second, the use of Paxlovid - at least in Germany - is rather limited and below expectations, third, Paxlovid was approved after the Omicron variant started to spread, and a new model is required to accommodate the Omicron variant.

15. Methods, line 86: if more than one country is selected, how is migration handled? Can more than one country be selected with further location disaggregation by region with urban and rural specification?

The model is not limited to the number of locations considered, or the level of disaggregation assumed. However, the age-dependent contact behavior between and within the locations should be adequately captured, and this is where practical problems arise. Obviously, the number of compartments is increasing substantially as more locations are selected. Hence, ultimately one reaches the limits of computational feasibility. Estimates for contact matrices exist for many countries. However, it is much more difficult to get age-stratified estimates on the mobility behavior.

16. Methods, line 93: can the authors describe (if not done so in the text beyond this point) or elude to here that this will be further described, what the profile of emerging new variants that become dominant looks like in a given population/location?

We now specify that this is described later and in the mathematical details. We feel further description at this point would cause unnecessary repetitions.

17. Methods, line 98: in the late-infectious period, have symptoms, if any presented, been reduced or is the individual assumed to be asymptomatic during this stage?

Actually, this is irrelevant for the model dynamics. Distinguishing between asymptomatic and symptomatic infections here, is just to determine which infections might become isolated, and which infections are potentially lethal. Definitely, those that lead to recovery become asymptomatic in practice, while those which are lethal remain symptomatic until death. We made it clear, now.

18. Methods, line 105: can the authors please provide a rationale for why they chose to assume people who have been infected become permanently immune? This does not reflect reality. Immunity wanes and people can become reinfected.

The rational is lengthy. The model, at the time of submission, was modelling the pandemic before the occurrence of the Omicron variant. In most countries, where such a model could be parametrized due to sufficient epidemic monitoring, only few viral variants were circulating and incidence was relatively low. Hence, reinfections could be safely neglected. This is no longer true with the Omicron variant that requires a completely new model. However, before Omicron it was a safe assumption, as the acquired immunity has a long-lasting effect (immunity declines modestly after 6 to 8 months, see Danetal2021).

19. Methods, line 120: following successful immunization, is there immunity waning? If so, what is the profile of the decay (e.g., exponential or biphasic, with a specified half-life, etc.)

Full immunity following successful immunization is assumed to be permanent, waning immunity and reinfection are not modeled. At the time of submission, booster campaigns just started during the delta wave, with very high propensity to receive a booster shot. It was with the spread of the Omicron variant with milder episodes that vaccination hesitancy increased. So, at the point we submitted the model, this was a reasonable assumption, which is no longer true at the current state of the pandemic.

20. Methods, line 122: Can the authors state whether all necessary dose with a respective vaccine product represents primary series (i.e., one or two doses) or also booster doses (and if so, how many booster doses – it is only noted on line 482 that booster shots can be implemented, this should be made clear earlier)? Also, since doses are given only one time, that this does not represent the real-life peak and decay of vaccine-induced immunity, and the implications this simplification will have on projections.

For each vaccine, doses are subsumed in the waiting times for the outcome to manifest. Incorporating booster vaccines also requires modeling immune waning (see comment 19). This is a completely new model. We clarified that the vaccination schedule is subsumed by the rates at which the effect manifests.

21. Methods: As an overall suggestion, reference to relevant sections from the Mathematical Appendix could be referred to appropriately throughout the Methods section.

We thank the reviewer for the suggestion. This is adopted for the necessary formulas.

22. Methods, line 141: are the proportion of those symptomatic further disaggregated into clinical and severe (prior to being lethal)?

Such a fine-grained distinction is not necessary, as it can be modelled as a multiplicative factor.

23. Methods, line 146: this sentence should be reworded something like 'Individuals can get infected during the time when the outcome of the vaccine is still pending.' As written, the comma between the two clauses of the sentence makes interpretation unclear.

Done.

24. Methods, line 149: again, is there decay following partial immunity?

See point 19

25. Methods, line 157: is the period during which that outcome of the vaccine is still pending define by the user, or is this specified in the model similarly for all vaccine products?

The waiting time for the vaccine outcome to manifest is a parameter, which reflects the vaccination schedule and the time the vaccine needs to develop its full effect, e.g., 7 days after the second dose. The parameter should be carefully selected after consulting the clinical studies of the vaccine products and the recommended vaccination schedule (this varies between countries - e.g. for the BioNTech the recommendation is to have the two doses 2-4 weeks apart). Since this is mentioned now at a different place (see point 20.) we did not want to repeat it.

26. Methods, lines 161-162: the punctuation needs to be corrected to 'immunization (partially-immune are less likely to get infected than non-immunized susceptibles).' the first period was omitted and a close bracket was inserted before the second period.

Done.

27. Methods, line 168: can the authors elaborate on how locations are defined, e.g., household, community, school, workplace or other location? Are there probabilities of transmission for each location, it is assumed so, are these listed anywhere? Later in the Results section 'Population and contact behavior' the locations are described, this seems to be better suited for description in the Methods section.

We state now that we use Prem matrices for the contact behavior. This is just a pragmatic choice, because it allows an intuitive parametrization of contact reductions. Contact matrices at a fine-grained level and the contact behavior between locations is difficult in practice, because estimates of mobility are difficult.

28. Methods, line 173: perhaps the authors could add a sentence describing the effect seasonality plays on basic reproduction numbers, for example, during warmer seasons (spring and summer) individuals tend to remain outdoors more, therefore contact numbers are reduced and basic reproduction is decreased by a given percentage, with the opposite assumed for cooler seasons.

This is a good idea, but the explanation is actually found in the appendix. We added a sentence, but intentionally kept it at a minimum to avoid unnecessary repetitions.

29. Methods, line 180: how long after the start of the epidemic is it assumed that case isolation effect will be modelled?

It is important to keep the general model description strictly separated from the particular application to Germany, or our specific parameter choices. We added a statement, clarifying that case isolation can stop at any time point.

We tried to present the model description in a very structured way. We avoided lengthy explanations, because with this model complexity, too many detailed explanations could jeopardize the readability. We therefore aimed for a balanced compromise.

30. Methods, line 182: following convention used by the WHO, social distancing should be referred to as physical distancing.

We agree! Done.

31. Results, line 203: is this population size representative of the population of Germany for the year 2020 for example? Please clarify.

The demographic data used for Germany are extracted from census data from the year 2020. This is approximate, depending on the precise census point of the demographic data. The exact values change - it depends on micro and macro census, citizenship, residence status, etc. Anything between 82 - 84 million will be sufficiently accurate. This information is backed up in the manuscript by a reference to the "Federal Statistics office of Germany".

32. Results, line 213: the authors state that 'estimates for mobility were unavailable to us', could not the Google Mobility data (as noted on line 438), which was available prior to November 2021, have been used? This could have been adapted to the contract matrix design. Later on lines 442-442, the authors state that 'Because adequately estimating mobility data would have, exceeded the scope of this article. This should be stated earlier.

We definitely tried to use that. However, the data is useless for our purposes because the information is not age-stratified. Hence, one cannot construct a contact matrix from this data. Actually getting good mobility estimates would not just be a completely new manuscript, but a whole multi-year research endeavor.

33. Results, line 220: are these 't' values reported in number of days in the burn-in period? Please include units. The same applies for this instance on line 227, and throughout.

Units are added. "t" is time since the start of the outbreak "t=0".

34. Results, lines 221-222, S4 table: the title should reflect that these parameters where assumed for Germany, if true. For the asterisk definition of incidence, it would be useful to state if these are (confirmed and unreported) SARS-CoV-2 cases. As well for 'per 100 000' note that these are individuals. Is there a reference to support how incidence thresholds were set?

This was clarified. There is a reference in the Mathematical appendix. We assumed that about 50-60% of the infections are detected. Hence, we adjusted the incidence based thresholds accordingly. Several provinces in Germany had similar rules in addition to the federal emergency brakes. Du to a lengthy list of rather obvious problems, these incidence-based thresholds are not perfect. I.e., there is a lack of reporting and actions are not instantaneous, as they differ between counties etc. We feel that a lengthy discussion would be out of place.

35. Results, line 224: by convention variant names 'Alpha variant' are not hyphenated as was done here.

Done.

36. Results, line 228: since the Greek symbol superscript was used for Delta on line 230, for consistency, the Greek symbol for Alpha should be used here vs 'A'.

The Greek capital Alpha looks just looks like an "A". This might look different in the font of the printed version, but there is nothing we can do about it.

37. Results, line 232: is the 43% seasonality fluctuation defined based on a reference(s)? For December 21, is the year 2021?

The value of 43% is chosen according to known fluctuations of R0 in influenza. This is a standard value. We discuss it more in one of our other models (Tsounguietal2021). Notably, the 43% apply to Germany, it has to be chosen differently across countries. It is essentially motivated by fluctuations in UV radiations. Time t=300 corresponds for Germany to December 21, 2020. Everything is clarified now.

38. Results, line 238: it would be helpful to clarify how adjustments were made for weather, i.e., contact increases due to colder weather where individuals tend to remain indoors more, even if this is further described in S1 of the appendix.

We agree with the reviewer and have added a statement.

39. Results, lines 240-241: include the type of virus for each vaccine product.

We wanted to avoid a lengthy discussion. Precise estimates are notoriously difficult. We used values which were estimated in the initial clinical studies. However, the study design differed across the manufacturers. Also, only the efficacy is reported, not the effectiveness. Hence, a one-to-one comparison is difficult. We consider the estimates common knowledge. For certain combinations, one needs to be careful: e.g., the AstraZeneca vaccine has low efficacy on the Beta variant.

40. Results, line 242: was the vaccine given to 60+ (the oldest age group) or was priority in Germany first targeted at even more at risk, e.g., 80+ or those with comorbidities or in long-term care in Germany? If this differs to the historical real-life strategy, state how this might have affected the pre-simulation fitting, etc.

This is an irrelevant factor for Germany, because incidence was so low in the high risk group, and vaccination deployment so fast, that this fine-grained differentiation will not make a big difference. Clearly, one could use more age strata than we did. If good parameter estimates are available, this will lead to more efficient predictions. The typical problem are the different interval scales reported in across different sources, e.g., age groups of 5 years vs. age groups by educational levels (<5, 6-10, 10-14, 14-18,19-65, 65+). Those are all well-known problems, and we believe it has no value to discuss it in detail.
With rather intuitive parameters, the model fit is quite good. The purpose of the ms is to introduce the model, not the data application.

41. Results, line 259: have 'U, V', Im, PI, NI' been previously defined?

Agreed. We fixed it and introduced the abbreviations in the Methods section.

42. Results, line 268: can a reference for the dynamics in Germany be cited?

Done.

43. Fig 3 caption: define 'E, P, I, L'.

Done.

44. Results, line 273: remove the duplicate 'the'.

Done.

45. Results, 279: it would be useful to state what the seasonal fluctuation were. Late August 2021 was unusually cold, but what was the weather pattern in August 2020?

This is subsumed with the weather adjustments. We refer to this section now.

46. Results, 281: perhaps instead note end of December 2021 during the Christmas holiday, and make it explicit that this peak occurred at the end of December because thereafter stricter measures where instated.

Done.

47. Results, line 288: this should be ‘increased again as of mid-October’.

Done.

48. Results, lines 290-291: state why infections among children and teens were higher in the 3rd and 4th waves vs 1st and 2nd waves, e.g., because of vaccine eligibility? Similarly for the incidence among <5 in the 4th wave vs teens.

This is a good idea, we added a statement and references.

49. Results, lines 296-297: is there a published strategy (press release, report) than can be cited for Germany?

Done.

50. Results, line 297: did vaccination rates increase in only the 60+ group here, or was eligibility already extended to younger groups (hard to discern as there is no time period specified)?

References were added. Again, this is not too important. In general, the supply of the vaccine increased over time and so the rates to get vaccinated. There were numerous exceptions to the prioritization. It is impossible to get exact numbers. At a certain point, the vaccination rates do not matter, once everybody willing to get vaccinated actually got vaccinated.
Vaccination rates increased while keeping the priority on the risk groups and healthcare workers. This information is not explicitly described in the manuscript, as we are trying to keep it short and concise. However, the information is described in the references we added. See point 49.

51. Results, line 300: here ‘Johnson & Johnson’ was used, but on lines 241 and 243, no spaces were used for Johnson&Johnson, make consistent.

Done.

52. Results, line 301: add ‘of’ before ‘2021’. This timing and coverage level should be stated earlier or this section referenced above (see comment above).

“Of” was added. We disagree with making the statement earlier. For the intro this information would be too specific, in the methods we concentrate on the general description of the model, and in the results, which are specific to the application to Germany, the information was withheld in the first section describing the parameters because it would be too detailed and too detached from the actual description of the dynamics.

53. Results, line 304: reword as ‘during the 2021 flu season’

Done.

54. Results, line 307: correct the typo ‘form’ to ‘from’

Done.

55. Results, line 308: replace ‘hypothetical’ with ‘counterfactual’

Done.

56. Results, line 309: what period does ‘current’ represent, November 22, 2021 the start of the simulation period? And should it be ‘i.e., schools remained closed’

Here, `current' refers to November 22, 2021. This was made explicit In the manuscript.

57. Results, line 311: on line 286, it is stated that a fourth wave started late August 2021, are the authors predicting that this wave will last until May 2022 (~8 months+) or am I misunderstanding?

This is correct, we made it explicit. By now we know that stronger contact reductions were in fact implemented in December 2021 after the ms was submitted, and that these relatively strong interventions led to a decline in incidence during a wave which was substantially higher as the second wave. However, the arrival of the Omicron variant yielded a new, much stronger wave. The model as stated above is no longer adequate for the omicron wave. Also, the data in spring 2022 can no longer be trusted. Namely, incidence declined exactly by the same amount as COVID-tests declined. The positive rate remained fairly stable.

58. Results, line 313: it would be useful to quantify the rough peak estimate for incidence, in addition to stating that it would be 7x-higher than for the 2nd wave. The same for this convention on lines 316-317.

We added the 7-day incidence, however, it includes the unreported cases, which we assume to be between 40-50% of the total cases.

59. Results, line 324: it is rather schooling from home (i.e., via Zoom by teachers) vs home schooling (which implies lessons are given by parent(s))

Done.

60. Results, line 333: add a comma after ‘approved’

Done.

61. Results, line 346: are the authors suggesting mandatory vaccination for individuals who reside in Germany? Would there still be an influence from immigration, or would anyone entering the country also have to be vaccinated?

This is an academic question. In practice, it cannot be controlled. There will always be border traffic via trains and cars, which is not as easily controlled as air traffic. In the model, there are always external infections assumed. By controlling the number of external infections, one accounts for the vaccination coverage of tourists, etc. Discussing this more would mean getting lost in unnecessary details.

62. Results, line 349: how relatively short is this period? What is the daily vaccination rate?

This is tricky, because it requires a reparametrization of the model, as the number of individuals who are unvaccinable increases. Therefore, the vaccination rates need to be parametrized in a way that the vaccination progress resembles the empirical observations. For the particular application to Germany, the vaccination rate after a mandatory vaccine is irrelevant, because of the relatively long vaccination schedule compared with the proximity to the epidemic peak. Nevertheless, the parameters are given in the supplementary tables, to which we refer now. Also, the vaccination coverage was already high. We feel that Figure 7D explains everything already and that a detailed discussion would be out of place.

63. Results, lines 350-351: vaccination may be much less effective than school closures or an emergency brake from these modelled findings, but it is important to state that NPIs cannot be maintained indefinitely and there are economic and social implications, which can be avoided through vaccination.

Agreed. This clarification was added to the manuscript.

64. Results, line 359: replace ‘mutation’ with ‘variant’, as there are >1 mutations, i.e., N501Y, A570D, etc.

Done.

65. Discussion, lines 367-368: this may have been true at the time of writing, but many governments have now relaxed measures – this should be reflected prior to publication.

We modified the sentence appropriately.

66. Discussion, line 374: the wording ‘do not everywhere progress’ is awkward

Done.

67. Discussion, line 374: what is the definition for under-aged, not eligible for vaccination, i.e. <5?

The sentence was revised.

68. Discussion, line 391: revise to ‘that will occur in the future’

Done.

69. Discussion, line 392: are individual mutants captured in the model or new variants of concern?

The model is not limited to certain mutations. However, with too many variants, the dimension of the system increases substantially. Hence, it is appropriate to bundle some variants with similar properties. Until the occurrence of the Omicron variant, this was not a problem anyway.

70. Discussion, lines 400-401: how do (1) effectiveness and (2) protectiveness from infection and transmission differ?

The effectiveness of the vaccines refers to their ability to protect the vaccinated individuals from severe disease (i.e., that corresponds mainly to the clinical efficacy studies), while protectiveness is used to define the ability of the vaccines to limit the spread of the viruses from a vaccinated and reinfected individual. This is clarified now.

71. Discussion, line 402: revise to ‘not all vaccines are equally’ and close brackets after reference 13, and the last part of this sentence does not make sense, do the authors mean ‘do not fully protect’?

Done.

72. Discussion, lines 403-406: it seems a bit out of scope for a modelling paper to discuss why vaccines do not fully protect. If this is retained, neutralizing antibodies, memory cells, CD4 T cells, etc. This is complex and not fully understood.

The immune response is a complex story. However, it is not proven that the vaccines lead to IgA production and the argument is intuitive. The statement is formulated as a possible explanation, not as empirical evidence. We placed this statement intentionally, based on our interaction with other mathematical modelers. Initially, most of them - despite empirical evidence - were convinced of the transmission-reducing effect of the vaccine. We wanted to give an intuitive explanation, of why it cannot be expected, that the vaccine protects from infection. Namely, the COVID vaccine was compared to the measles vaccine, usually for good reasons. The virus has the same routes of transmission and is similarly contagious. However, measles replicate in the lymph nodes, which is very different from SARS-CoV-2.

73. Discussion, line 458: insert ‘cold weather’ in front of ‘extremes’

Done.

74. Discussion, lines 467, 469, 471: define to what ‘currently’ refers

The term “currently” refers to the end of November 2021. We made that explicit now.

75. Discussion, line 469: consider adding ‘and subregion’ after ‘to any country’

Done.

Reviewer #2: Predicting the impact of COVID-19 vaccination campaigns - a flexible age-dependent, spatially-stratified predictive model, accounting for multiple viral variants and vaccines
The subject is interesting, but the authors must correct and explain these points:
Title
1. The title is not informative. Consider revising it.

We changed the title as follows: “A flexible age-dependent, spatially-stratified predictive model for the spread of COVID-19, accounting for multiple viral variants and vaccines”.

Abstract
1. Background: The study question is not mentioned, and the study's rationale is obscure. Please make the objective of the study crystal clear, using a sentence beginning with "the objective of this study was to …".

The objective was made clear now.

2. Conclusion: Considering that the title presents the study question and the conclusion presents the answer the authors found for that question, the conclusion needs to be re-written. The conclusion needs to be inevitably derived from the results section.

We thank the author for this reminder of the structure of scientific manuscripts, in addition to our well-established publication records. However, we have to disagree. The objective is to develop a model that can be used as a tool to quantify the dynamics of the COVID-19 epidemic. The main intellectual contribution is the setup of the model, which is presented in the mathematical appendix. The results are an example of the applicability of the model and have their own intellectual value. It is not new that the structure of scientific articles, which increasingly standardized during the last 10 years, is not optimal for the type of work presented here.

Introduction
1. A paragraph is missing between lines 51-52 to present the existing gap in the literature.
We inserted a sentence stating the essence of the missing gaps in the literature. Namely, most mathematical models are too unrealistic to produce appropriate predictions, while they focus on mathematical techniques such as deriving a stability analysis of a disease-free equilibrium, which are inapplicable and irrelevant for the current epidemic management. We point out that the manuscript was submitted in November 2021, when the lack of realistic models accounting for the effects of the COVID-19 vaccines was obvious. We felt then and feel now that a lengthy discussion on the literature of mathematical models would be misplaced, uninteresting for the targeted readers, and highly distracting in the manuscript.
Methods
1. Please include an overview at the beginning of the methods section.

We believe the method section has an overview. Nevertheless, we added some more lines.

Discussion
1. Please include the model's implications at the beginning of the discussion section.

We intentionally avoided doing so for several reasons: 1. The ms is written for a broad readership. They do not read it sequentially but read the abstract, the intro, and maybe the discussion before going into the details of the results and methods - not to speak of the mathematical details. 2. The implications are rather straightforward: the vaccine is beneficial for the control of the epidemic dynamics. However, the real merit of the model lies in its ability to quantify this effect. Starting a discussion with the implications of such a technically involved and complex model would only be confusing. We believe it is more important to capture the relevant context (at the time of initial submission) before discussing the model. 3. By starting with the implications, the discussion will necessarily read repeatedly, because we would first need to summarize the results, then discuss their relevance in the appropriate context, and then go back to technical details of the model.
Hence, although every PhD student learns in scientific writing 101 to put a little summary in a discussion, it is a judgment of scientific maturity and decades of experience to depart from these rules of thumb.
